# Supplementary material for: Warmer and wetter conditions will reduce offspring production of hawksbill turtles in Brazil under climate change
Source: PLoS One. 2018 Nov 8;13(11):e0204188. doi: 10.1371/journal.pone.0204188 (PMC6224045; doi:10.1371/journal.pone.0204188)
Supplement: S1 File — Distances of INMET weather stations from nesting grounds in each region considered in this study, from north to south (Table A). Nest and climate data availability for each region between 2005–2016 included in our analyses. The typical nesting season in RN occurs between November–May, while the typical nesting season in BA occurs between October–April (Table B). Results of Tamhane’s T2 test for statistical differences in hatching success and emergence success between nesting beaches within Rio Grande do Norte (RN) and Bahia (BA), Brazil, from north to south. Statistically significant p–values are indicated in bold (Table C). Results of Generalized Linear Mixed-Effects Models for local climate influences on hatching success (HS) and emergence success (ES) across Brazil as well as within Rio Grande do Norte (RN) and Bahia (BA). For these models, the binomial family was specified, and the year nests were laid was the random effect. Model parameters included: average air temperature (temp), accumulated precipitation (acc.rain), average precipitation (avg.rain), average humidity (humid), average solar radiation (rad) and average wind speed (wind). The temporal scales used were: the month nests were laid (0.climate variable), the month nests were laid and one-month prior (0.1.climate variable), the month nests were laid and two months prior (0.2.climate variable), two months prior to nesting (2.climate variable), and during the incubation period (inc.climate variable). The models with the lowest AICc values and high significance are highlighted in gray. P–values for combined models are presented for each parameter in the order the model is written (Table D). (PDF) [file pone.0204188.s001.pdf]

Warmer and wetter conditions will reduce offspring production of hawksbill turtles in Brazil under climate change

Natalie Montero[1], Maria A.G. dei Marcovaldi[2], Milagros Lopez–Mendilaharsu[2], Alexsandro S. Santos[2], Armando José Barsante Santos[2], Mariana M.P.B. Fuentes[1]*

[1] Department of Earth, Ocean, and Atmospheric Science, Florida State University 117 N Woodward Avenue Tallahassee, Florida, USA

[2] Fundação Pró-Tamar, Rubens Guelli, 134, sala 307, Salvador, Bahia, Brazil

Table A.

Weather Station	Nesting Beach	Distance (km)
Natal, Rio Grande do Norte	Cacimbinhas	49
	Madeiro	52
	Chapadao	55
	Minas	53
	Sibauma	60
Salvador, Bahia	Imbassai	79
	Praia do Forte	69
	Arembepe	41
	Santa Maria	34
	Busca Vida	28

Table B.

Rio Grande do Norte months considered (n = 66)	Bahia months considered (n = 67)
Nov 2005 – Apr 2006; Dec 2006 – May 2007; Nov 2007 – Apr 2008; Nov 2008 – Apr 2009; Nov 2009 – Mar 2010; Nov 2010 – Apr 2011; Dec 2011 – Apr 2012; Nov 2012 – May 2013; Nov 2013 – May 2014; Dec 2014 – Apr 2015; Nov 215 – May 2016	Oct 2005 – Dec 2005; Oct 2006 – Mar 2007; Oct 2007 – Jan 2008; Oct 2008 – Apr 2009; Oct 2009 – Apr 2010; Oct 2010 – Apr 2011; Oct 2011 – Apr 2012; Nov 2012 – Apr 2013; Oct 2013 – Mar 2014; Oct 2014 – Apr 2015; Oct 2015 – Apr 2016

Table C.

Region	Nesting Beach	Hatching Success	Emergence Success
RN	Cacimbinhas – Madeiro	0.073	0.259
	Cacimbinhas – Chapadao	**0.000**	**0.000**
	Cacimbinhas – Minas	**0.000**	**0.000**

	Cacimbinhas – Sibauma	0.596	0.825
	Madeiro – Chapadao	**0.000**	**0.000**
	Madeiro – Minas	**0.000**	**0.000**
	Madeiro – Sibauma	0.052	0.273
	Chapadao – Minas	0.241	0.407
	Chapadao – Sibauma	**0.019**	**0.004**
	Minas – Sibauma	0.206	0.014
BA	Imbassai – Praia do Forte	**0.000**	0.887
	Imbassai – Arembepe	**0.000**	**0.000**
	Imbassai – Santa Maria	0.342	0.427
	Imbassai – Busca Vida	0.233	**0.009**
	Praia do Forte – Arembepe	**0.000**	**0.000**
	Praia do Forte – Santa Maria	**0.000**	0.622
	Praia do Forte – Busca Vida	**0.000**	0.017
	Arembepe – Santa Maria	**0.000**	**0.000**
	Arembepe – Busca Vida	**0.000**	**0.000**
	Santa Maria – Busca Vida	**0.002**	0.146

Table D.

Model	Brazil HS		RN HS		BA HS		Brazil ES		RN ES		BA ES	
	**AICc**	**p-value**	**AICc**	**p-value**	**AICc**	**p-value**	**AICc**	**p-value**	**AICc**	**p-value**	**AICc**	**p-value**
0.temp	186250.4	<2 e-16	52499.71	82589.3	<2 e-16	3.56 e-15	82589.3	<2 e-16	22573.67	1.86 e-8	57494.39	<2 e-16
0.1.temp	186486.2	<2 e-16	52557.88	82218.41	<2 e-16	0.039	82218.41	<2 e-16	22536.56	<2 e-16	57434.21	<2 e-16
0.2.temp	186562.3	0.839	52555.74	81690.16	<2 e-16	0.011	81690.16	<2 e-16	22390	<2 e-16	57262.46	<2 e-16
2.temp	186517.9	2.47 e-11	52503.05	81502.04	<2 e-16	1.15 e-14	81502.04	<2 e-16	22178.98	<2 e-16	57231.1	<2 e-16
Inc.temp	183339.6	<2 e-16	51816.73	82911.09	0.0406	<2 e-16	82911.09	0.0406	21751.55	<2 e-16	56910.28	<2 e-16
0.acc.rain	185659.1	<2 e-16	52319.96	81639.76	<2 e-16	<2 e-16	81639.76	<2 e-16	22148.54	<2 e-16	57501.32	<2 e-16
0.1.acc.rain	185012.6	<2 e-16	52293.84	81765.43	<2 e-16	<2 e-16	81765.43	<2 e-16	22179.32	<2 e-16	57525.58	<2 e-16
0.2.acc.rain	184864.4	<2 e-16	52277.17	82010.81	<2 e-16	<2 e-16	82010.81	<2 e-16	22068.01	<2 e-16	57543.65	6.72 e-15
2.acc.rain	185669.8	<2 e-16	52426.6	82768.4	<2 e-16	<2 e-16	82768.4	<2 e-16	22336.64	<2 e-16	57596.5	0.00318
Inc.acc.rain	186150.9	<2 e-16	52154.22	80483.78	<2 e-16	<2 e-16	80483.78	<2 e-16	21927.53	<2 e-16	56859.93	<2 e-16
0.avg.rain	185892.6	<2 e-16	52165.9	81895.67	<2 e-16	<2 e-16	81895.67	<2 e-16	22012.1	<2 e-16	57505.05	<2 e-16
0.1.avg.rain	185066.2	<2 e-16	52141.97	81708.88	<2 e-16	<2 e-16	81708.88	<2 e-16	22013.81	<2 e-16	57487.77	<2 e-16
0.2.avg.rain	185082.5	<2 e-16	52159.63	81975.57	<2 e-16	<2 e-16	81975.57	<2 e-16	21954.44	<2 e-16	57500.61	<2 e-16
2.avg.rain	185904.9	<2 e-16	52393.48	82757.36	<2 e-16	<2 e-16	82757.36	<2 e-16	22287.66	<2 e-16	57597.77	0.000251

Inc.avg.rain	186192.8	<2 e-16	52194.13	82888.01	5.22 e-6	<2 e-16	82888.01	5.22 e-6	21916	<2 e-16	57592	0.000956
0.humid	185042.9	<2 e-16	52322.71	82486.37	<2 e-16	<2 e-16	82486.37	<2 e-16	22186.01	<2 e-16	57604.57	0.412
0.1.humid	185164.7	<2 e-16	52292.86	82806.12	<2 e-16	<2 e-16	82806.12	<2 e-16	22199.49	<2 e-16	57579.08	2.95 e-7
0.2.humid	185271.6	<2 e-16	52298.42	82866.81	2.71 e-12	<2 e-16	82866.81	2.71 e-12	22128.09	<2 e-16	57582.51	1.73 e-6
2.humid	185882.9	<2 e-16	52404.62	82910.57	0.0242	<2 e-16	82910.57	0.0242	22323.51	<2 e-16	57555.38	1.48 e-12
Inc.humid	185650.1	<2 e-16	52047.42	81114.97	<2 e-16	<2 e-16	81114.97	<2 e-16	21706.38	<2 e-16	57360.67	<2 e-16
0.rad	184888.7	<2 e-16	52470.36	82089.81	<2 e-16	<2 e-16	82089.81	<2 e-16	22315.88	<2 e-16	57306.44	<2 e-16
0.1.rad	184874.1	<2 e-16	52465.02	82800.2	<2 e-16	<2 e-16	82800.2	<2 e-16	22400.85	<2 e-16	57592.38	0.00034
0.2.rad	185664.7	<2 e-16	52404.04	82911.32	0.0371	<2 e-16	82911.32	0.0371	22407.61	<2 e-16	57587.61	2.62 e-5
2.rad	186402.9	<2 e-16	52498.06	82594.68	<2 e-16	9.73 e-16	82594.68	<2 e-16	22568.62	8.63 e-10	57375.08	<2 e-16
Inc.rad	184190.1	<2 e-16	51885.58	79958.17	<2 e-16	<2 e-16	79909.3	<2 e-16	21582.37	<2 e-16	56194.78	<2 e-16
0.wind	186470.2	<2 e-16	52045.63	82556.38	<2 e-16	<2 e-16	82556.38	<2 e-16	22057.66	<2 e-16	57570.32	2.94 e-9
0.1.wind	186506.6	7.46 e-14	52170.94	82501.82	<2 e-16	<2 e-16	82501.82	<2 e-16	22115.43	<2 e-16	57549.07	4.47 e-14
0.2.wind	186502.6	9.74 e-15	52125.11	82491.03	<2 e-16	<2 e-16	82491.03	<2 e-16	22111.55	<2 e-16	57533.77	<2 e-16
2.wind	186519.8	6.25 e-11	52348.91	82465.74	<2 e-16	<2 e-16	82465.74	<2 e-16	22370.81	<2 e-16	57533.93	<2 e-16
Inc.wind	186562.3	0.786	52388.38	82839.75	4.25 e-10	<2 e-16	82839.75	4.25 e-10	22032.03	<2 e-16	57593.34	0.00162

Inc.temp + 0.acc.rain	182977.8	<2 e-16; <2 e-16	51805	81641.77	0.99; <2 e-16	<2 e-16; 0.0002	81641.77	0.99; <2 e-16	21679.77	<2 e-16; <2 e-16	56911.99	<2 e-16; <2 e-16
Inc.temp + 0.1.acc.rain	182679.5	<2 e-16; <2 e-16	51816.25	81766.24	0.285; <2 e-16	<2 e-16; 0.114	81766.24	0.285; <2 e-16	21718.82	<2 e-16; 3.7 e-9	56911.89	<2 e-16; 0.532
Inc.temp + 0.2. acc.rain	182508.5	<2 e-16; <2 e-16	51818.54	82010.59	0.148; <2 e-16	<2 e-16; 0.653	82010.59	0.148; <2 e-16	21708.57	<2 e-16; 1.94 e-11	56901.68	<2 e-16; 0.00117
Inc.temp + 2.acc.rain	182869.2	<2 e-16; <2 e-16	51801.68	82764.81	0.0243; <2 e-16	<2 e-16; 3.56 e-5	82764.81	0.0243; <2 e-16	21750.87	<2 e-16; 0.101	56902.26	<2 e-16; 0.00159
Inc.temp + inc.acc.rain	183292.3	<2 e-16; 2.5 e-12	51801.68	80485.62	0.684; <2 e-16	<2 e-16; 0.809	80485.62	0.684; <2 e-16	21713.93	<2 e-16; 1.9 e-10	56653.11	<2 e-16; <2 e-16
Inc.temp + 0.avg.rain	183117.1	<2 e-16; <2 e-16	51738.15	81891.39	0.0174; <2 e-16	<2 e-16; <2 e-16	81891.39	0.0174; <2 e-16	21603.56	<2 e-16; <2 e-16	56906.54	<2 e-16; 0.0168
Inc.temp + 0.1.avg.rain	182788.7	<2 e-16; <2 e-16	51777.82	81709.01	0.184; <2 e-16	<2 e-16; 1.56 e-10	81709.01	0.184; <2 e-16	21654.99	<2 e-16; <2 e-16	56909.51	<2 e-16; 0.0966
Inc.temp + 0.2.avg.rain	182827.6	<2 e-16; <2 e-16	51807.78	81973.15	0.0443; <2 e-16	<2 e-16; 0.009	81973.15	0.0443; <2 e-16	21667.44	<2 e-16; <2 e-16	56897.6	<2 e-16; 0.000131
Inc.temp + 2.avg.rain	183113.7	<2 e-16; <2 e-16	51802.04	82753.32	0.0194; <2 e-16	<2 e-16; 4.32 e-5	82753.32	0.0194; <2 e-16	21749.83	<2 e-16; 0.0536	56902.92	<2 e-16;0.00226

Inc.temp + inc.avg.temp	183304.1	<2 e-16; 9.8 e-10	51816.2	81781.72	<2 e-16; <2 e-16	<2 e-16; 0.111	81781.72	<2 e-16; <2 e-16	21672.38	<2 e-16; <2 e-16	56911.75	<2 e-16; 0.476
Inc.temp + 0.humid	182720.5	<2 e-16; <2 e-16	51818.38	82485.17	0.0852; <2 e-16	<2 e-16; 0.552	82485.17	0.0852; <2 e-16	21716.62	<2 e-16; 1.18 e-9	56846.95	<2 e-16; 6.33 e-16
Inc.temp + 0.1.humid	182667.1	<2 e-16; <2 e-16	51817.93	82803.58	0.0414; <2 e-16	<2 e-16; 0.369	82803.58	0.0414; <2 e-16	21722.02	<2 e-16; 1.65 e-8	56810.88	<2 e-16; <2 e-16
Inc.temp + 0.2.humid	182693.5	<2 e-16; <2 e-16	51816.6	82863.84	0.0331; 2.2 e-12	<2 e-16; 0.144	82863.84	0.0331; 2.2 e-12	21720.52	<2 e-16; 8.18 e-9	56865.54	<2 e-16; 6.65 e-12
Inc.temp + 2.humid	182945.4	<2 e-16; <2 e-16	51809.78	82908.27	0.0466; 0.0283	<2 e-16; 0.0027	82908.27	0.0466; 0.0283	21746.03	<2 e-16; 0.00604	56895.58	<2 e-16; 4.25 e-5
Inc.temp + inc.humid	183244.3	<2 e-16; <2 e-16	51809.33	80359.07	<2 e-16; <2 e-16	<2 e-16; 0.0021	80359.07	<2 e-16; <2 e-16	21597.94	<2 e-16; <2 e-16	56897.31	<2 e-16; 0.000575
